# Effects of sub-chronic branched chain amino acid supplementation on markers of muscle damage and performance variables following 1 week of rigorous weight training

**DOI:** 10.1186/1550-2783-12-S1-P29

**Published:** 2015-09-21

**Authors:** Petey Mumford, Wesley C Kephart, Anna E McCloskey, Angelia M Holland, Joshua J Shake, C Brooks Mobley, Kaelin C Young, Jordan R Moon, Michael D Roberts

**Affiliations:** 1 School of Kinesiology, Auburn University, Auburn, AL, USA; 2 Edward Via College of Osteopathic Medicine - Auburn Campus, Auburn, AL, USA; 3 MusclePharm Sports Science Institute, Denver, CO, USA

## Background

We investigated the efficacy of supplementing a branched chain amino acid (BCAA- 3g/d L-Leucine, 1g/d L-Isoleucine and 2g/d L-Valine) supplement compared to a carbohydrate (CHO) control drink in terms of attenuating markers of muscle damage in addition to preserving performance markers following 3 days of intense weight training.

## Methods

Apparently healthy resistance trained males (n = 30) were randomized to either a BCAA group or the CHO control group. Participants performed preliminary testing (T1) to derive peak quadriceps isometric torque, peak quadriceps isokinetic torque (60º and 120º per second), and a 1RM barbell back squat. The following week, the participants performed 10x5 repetitions at 80% of their 1RM barbell back squat for 3 consecutive days. During this experimental intervention antecubital blood was drawn to assess serum myoglobin concentrations, in addition a visual analog scale was utilized in order to measure subjective perceptions of muscular soreness. 48 hours following the third bout of exercise, participants performed post testing (T2) like T1 testing and donated a final blood draw.

## Results

The BCAA group maintained 95% of their peak isometric torque compared to 86% for the CHO group (p = 0.12). Regarding isokinetic measures at T2, there was a 92% and 88% maintenance for the BCAA and CHO group, respectively (p = 0.39), compared to their respective T1 values. T2 performance at 120º/s was maintained by 93% and 96% of T1 measurements for the BCAA and CHO group, respectively (p = 0.40). The BCAA group actually enhanced squat 1RM by 1%, whereas the CHO group experienced a 3% decrement; however, this difference failed to reach significance (p = 0.92). Serum myoglobin concentrations increased as a function of time, and there was no difference between groups (p = 0.31). Lastly, perceptions of muscular soreness were also not differentially altered between groups (p = 0.09).

## Conclusions

In conclusion, while a BCAA supplement did not appear to enhance recovery benefits compared to a CHO control, a few areas of performance were bolstered to a point of practical importance regarding high level competition.

